# Effect of cerebral oxygen saturation on postoperative nausea and vomiting in female laparoscopic surgery patients

**DOI:** 10.1097/MD.0000000000008275

**Published:** 2017-10-13

**Authors:** WenJun Guo, Jie Ding, XiaoJu Jin, GaoJie Li

**Affiliations:** Department of Anesthesiology, Wannan Medical College, First Affiliated Hospital, Yijishan Hospital, Wuhu, Anhui, China.

**Keywords:** cerebral oxygen saturation, female, mannitol, postoperative nausea and vomiting

## Abstract

**Background:**

The purpose of this study was to investigate effect of cerebral oxygen saturation (SCTO_2_) on postoperative nausea and vomiting (PONV) in female patients who underwent laparoscopic surgery.

**Methods:**

This study included 90 female patients who underwent laparoscopic surgery (60 cases of gynecological operations and 30 cases of gallbladder operations). All patients were allocated into 3 groups of 30 patients each: group A (gynecological laparoscopic surgery), group B (gynecological laparoscopic surgery with mannitol treatment) and group C (laparoscopic cholecystectomy surgery). Perioperative SCTO_2_, mean blood flow velocity of vertebral artery (VM), vascular resistance index of vertebral artery (RI), and PONV (within 48 hours after surgery) were investigated.

**Results:**

No differences in age, body weight, operation time, and hemoglobin levels were observed among the patients (*P* > .05). The SCTO_2_ values for groups B and C were lower than those for group A in both brain hemispheres at T_4_ and T_5_ (*P* < .05). The VM was higher in group B than in groups A and C at T_3_ (*P* < .05), but differences in VM were not observed between groups B and C at T_4_ or T_5_. However, the VM of group A was still lower than the other groups (*P* < .05), and no difference in VM was observed among the 3 groups at T_6_ (*P* > .05). The RI was higher in group C than in groups A and B at T_4_ (*P* < .05). The incidence of PONV within 48 hours after surgery was significantly higher in group A than in the other 2 groups (*P* < .05).

**Conclusion:**

Strategies that maintain normal SCTO_2_ may reduce the incidence of PONV in female patients who underwent laparoscopy surgery by reducing perioperative intracranial pressure.

## 1 Introduction

As minimally invasive surgery and surgical techniques have improved, the number of gynecological laparoscopic surgeries has increased; however, the incidence of postoperative nausea and vomiting (PONV) is very high in patients undergoing gynecological laparoscopic surgery (53–72%).^[1]^ Acute postoperative vomiting may cause incision pain, the formation of a hematoma, or dehiscence of the incision, and an increased risk of inhalation pneumonia. All factors aforementioned not only increase the length of the hospital stay and financial burden but also reduce the patient's quality of postoperative recovery.^[2]^ The characters of this type of surgery included the Trendelenburg position and CO_2_ artificial pneumoperitoneum. The Trendelenburg position and intra-abdominal pressure lead to an intracranial venous obstruction and increased intracranial pressure. The Trendelenburg position increases the intracranial blood flow because of gravity and increased venous return resistance, thereby increasing the intracranial pressure. The abdominal laparoscopic operation requires CO_2_ to be pumped into the abdominal cavity to create space for the operation, resulting in elevated intra-abdominal pressure, generally 10 to 12 mm Hg. CO_2_ pneumoperitoneum may lead to hypertension because of hypercapnia. An increase in the intracranial blood CO_2_ concentration may result in intracranial vascular dilatation, subsequently leading to increasing intracranial pressure. The increased intracranial blood CO_2_ concentration indirectly increases the intrathoracic pressure and the blood flow resistance in the brain. These factors all increase the intracranial pressure. We hypothesize that the cause of the high incidence of PONV in patients who received gynecological laparoscopic surgery may be the high intracranial pressure that affects the uptake and utilization of oxygen by the brain tissue. The vestibular system, which is located in the brainstem, is very sensitive to ischemia, eventually leading to the occurrence of PONV.

The purpose of this study was to investigate the effect of cerebral oxygen saturation (SCTO_2_) in patients who underwent gynecological laparoscopic surgery in the incidence of PONV, then to study the incidence of PONV after using the mannitol to reduce intracranial pressure to verify the hypothesize. A reliable method for monitoring the intracranial pressure is currently unavailable; therefore, we examined the relationship between PONV that occurred within 48 hours after surgery and the following indicators: SCTO_2_, VM, and RI. We administered mannitol, a diuretic, before the operation to decrease the intracranial pressure and studied the changes in intracranial pressure and the changes in PONV in patients who underwent gynecological laparoscopic surgery. We then verified the relationship between high intracranial pressure and PONV in those patients.

## 2 Information and methods

### 2.1 Population and grouping

This study was approved by the medical ethics committee of our hospital, and informed consent was obtained from all patients.

Inclusion criteria: All patients were female. The patients’ ages ranged from 30 to 60 years, patients exhibited an American Society of Anesthesiologists (ASA) grade I to II physical status, and the body mass index (BMI) ranged from 18 to 24 kg/m^2^. The operation required over 50 minutes to complete and no longer than 120 minutes.

Exclusion criteria: Patients with a medical history of nausea and vomiting, hypertension, motion sickness, smoking, abnormal liver or kidney function, abnormal electrolyte levels, gastrointestinal disease, taking anti-emetic drugs before or after the operation, and cerebral disease were excluded from this study.

According to the simple random principle, the random numbers were got by computer. Ninety patients were divided into 3 groups using simple randomization. Thirty patients who underwent laparoscopic cholecystectomy and 60 patients who underwent gynecological surgery were allocated into 3 groups of 30 patients each: group A, patients who underwent gynecological laparoscopic surgery and were placed in the Trendelenburg position; group B, patients who underwent the gynecological laparoscopic surgery were placed in the Trendelenburg position and received the mannitol intervention (fast intravenous drip of 20% mannitol at 0.5 mg/kg prior to the operation); and group C, female patients who underwent laparoscopic cholecystectomy surgery and were placed in the reverse Trendelenburg position.

### 2.2 Methods

Patients were monitored with a 5 lead electrocardiogram (EKG), end tidal CO_2_ (PetCO_2_), a noninvasive blood pressure cuff for 3 minutes per measurement, and pulse oximetry (Ultraview SL 2700 Series Patient Monitor, Spacelabs Medical Inc, Issaquah, WA) routinely. The noninvasive cerebral oximetry (Fore-Sight, CAS Medical Systems, Inc, Branford, CT) was monitored to detect the changes in SCTO_2_ in the patients enrolled in study. Anesthesia induction: All patients received an intravenous injection of midazolam (0.05 mg/kg), sufentanil (0.5 μg/kg), propofol (1.5–2.0 mg/kg), and vecuronium (0.1 mg/kg). A laryngeal mask airway was placed and connected to an anesthesia machine (Anesthesia System Aesprie7900, Datex-Ohmeda Inc, Madison, WI) for intermittent positive pressure ventilation. Lactated Ringer solution was routinely intravenously dropped at a rate of 10 mL/kg.h, and anesthesia was maintained with propofol (6–8 mg/kg.h), remifentanil (0.15–0.20 μg/kg.min), and intermittent vecuronium as needed. PetCO_2_ was maintained between 35 and 45 mm Hg. Patient-controlled analgesia with 2.5 μg/kg sufentanil plus 100 mg of flurbiprofen was fixed when the skin was sutured. The pneumoperitoneum was generated with CO_2_ by the gas insufflator, and the pneumoperitoneum pressure was set in the machine. The pneumoperitoneum pressure of the 3 groups was set to 10 to 12 mm Hg. The noninvasive blood pressure fluctuated ± 20% compared to baseline values. Atropine and neostigmine were used to antagonize muscle relaxation. The laryngeal mask airway was removed when autonomous respiration was completely restored, and patients were sent to the ward 30 minutes later if their vital signs remained stable.

Use the Doppler (SonoScape S6 Ultrasound, SonoScape Medical Corp, Guangdong, China) to detect the VM and RI variations at each time point, the angle between the detector (3–5 MHz) and vessel was set to <60°. The following time points were used: T_1_, 5 minutes after entering the operation room; T_2_, 5 minutes after laryngeal mask airway placement; T_3_, pneumoperitoneum started and the patient's position changed; T_4_, 15 minutes after pneumoperitoneum with the position change; T_5_, pneumoperitoneum stopped with a position change; T_6_, 10 minutes after pneumoperitoneum had stopped. The SCTO_2_ value was also recorded at the same time points, and the value at T_1_ was the baseline value.

Because an accurate, noninvasive method for monitoring intracranial pressure is unavailable, the urine volume was used as an indirect index to reflect changes in intracranial pressure. The urine output measurement in group A and B.

PONV events that occurred within 48 hours were recorded. The PONV classification criteria were: I, no nausea and vomiting; II, 1 to 2 episodes; III, 3 to 4 episodes; and IV ≥5 episodes.

The drug labels were shaded. All monitoring procedures were performed by the same anesthetist who did not know the patient belonged to which group. PONV was investigated by another doctor who was blinded about the groups.

### 2.3 Statistical analysis

All data were analyzed using SPSS 19.0 software. Measured data are expressed as 
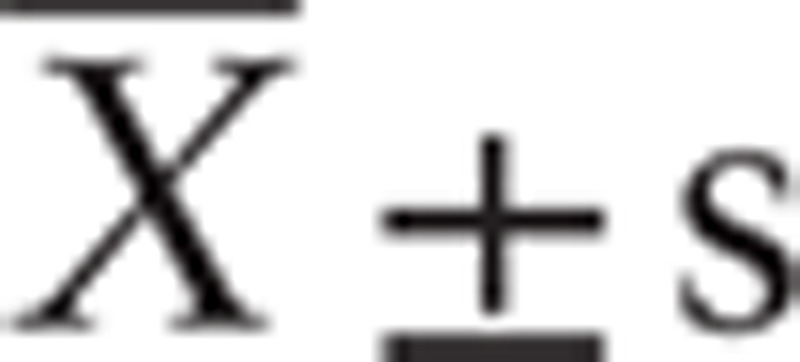
 ± s and comparisons among groups were performed using F and q tests. Enumerated data were analyzed using the χ^2^ test. *P* < .05 represents statistical significance.

## 3 Results

There were no significant differences in patients’ ages, weights, preoperative hemoglobin levels, and the operation times among 3 groups (*P* > .05) (Table 1).

**Table 1:**
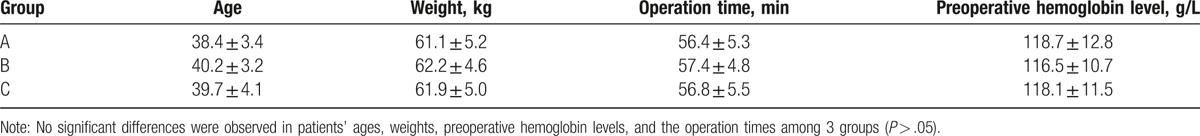
Basic information about the patients in the 3 groups.

**Figure 1:**
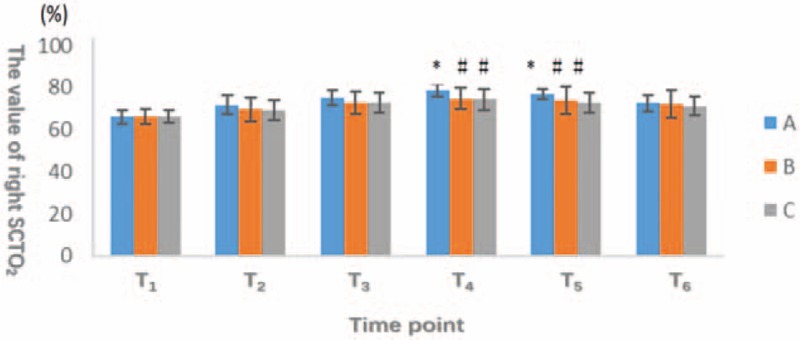
The horizontal axis represents different observation points, and the vertical axis indicates the number of SCTO_2_ of right and left sides of brain. The oxygen saturation of the patients in group A was significantly higher than that in group B and group C (*P* < .05) at the time of T_4_ and T_5_. There was no significant difference (*P* > .05) at other time points among 3 groups. SCTO_2_ = cerebral oxygen saturation.

**Figure 2:**
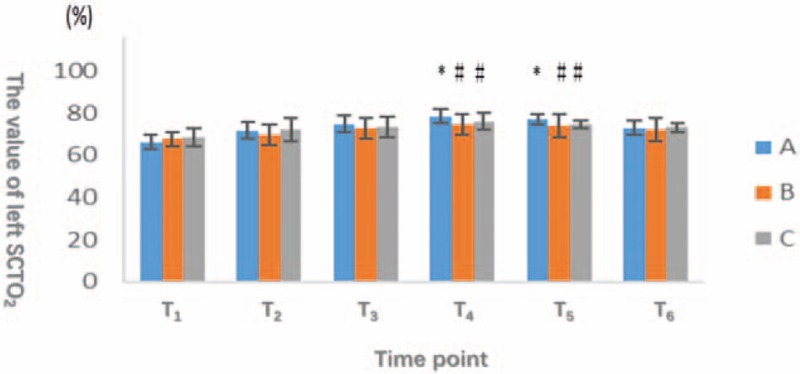
The horizontal axis represents different observation points, and the vertical axis indicates the number of SCTO_2_ of right and left sides of brain. The oxygen saturation of the patients in group A was significantly higher than that in group B and group C (*P* < .05) at the time of T_4_ and T_5_. There was no significant difference (*P* > .05) at other time points among 3 groups. SCTO_2_ = cerebral oxygen saturation.

At T_1_, T_2_, T_3_, and T_6_, we did not observe significant differences in the right or left SCTO_2_ (*P* > .05). At T_4_ and T_5_, both sides of the SCTO_2_ values were obviously decreased in groups B and C compared with group A, and this difference reached statistical significance (*P* < .05). However, the difference between groups B and C was not significant (*P* > .05) (Figs. 1 and 2).

From 15 minutes after beginning pneumoperitoneum and position change to 10 minutes after stopping pneumoperitoneum and postural recovery, the SCTO_2_ of group A was higher than the other 2 groups. Thus, the oxygen consumption by the brain tissue in group A was reduced.

**Figure 3:**
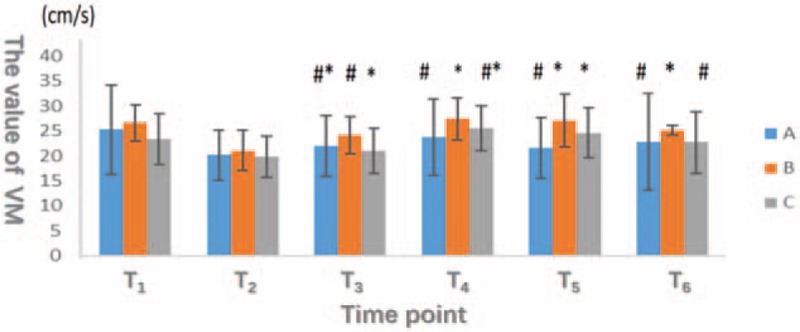
The horizontal axis represents different observation points, and the vertical axis indicates the value of VM (mean blood flow velocity of vertebral artery). The VM in group B is higher than group C at T_3_ (*P* < .05). The VM in group B is higher than group A at T_4_ (*P* < .05). The VM in group A is higher than group B and C (*P* < .05) and there is no significant difference between the group B and C (*P* > .05) at T_5_. The VM in group B is higher than group A and C at T_6_ (*P* < .05).

No significant difference in VM was detected at T_1_ and T_2_ among the 3 groups. At T_3_, VM was clearly higher in group B than in group C, and the difference reached statistical significance (*P* < .05). We did not observe a difference between groups A and C (*P* > .05); the data revealed a statistically significant difference between groups A and B at T_4_ (*P* < .05). At T_5_, VM was obviously increased in groups B and C compared with group A (*P* < .05); however, the differences between groups A, B, and C were not significant (*P* > .05). At T_6_, VM was still higher in group B than in groups A and C (*P* < .05), but the difference between groups A and C was not significant (*P* > .05; Fig. 3).

**Figure 4:**
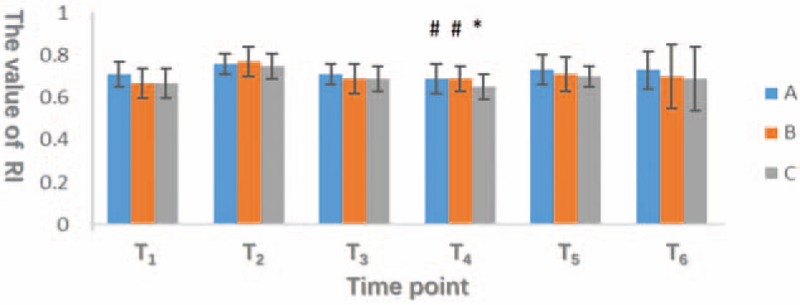
The horizontal axis represents different observation points, and the vertical axis indicates the RI (vascular resistance index of vertebral artery). The RI in group C is significantly lower than in group A and B at T_4_ (*P* < .05). There was no significant difference (*P* > .05) at other time points among 3 groups.

Differences in RI were not significant at T_1_, T_2_, T_3_, T_5_, and T_6_, whereas the RI of group C was significantly lower than the RI of the other 2 groups at T_4_ (*P* < .05; Fig. 4).

To summarize the results shown in Figures 3 and 4, the vertebral artery vascular resistance was significantly higher in group A than in the other 2 groups after pneumoperitoneum and postural changes; meanwhile, vertebral artery blood flow was obviously decreased in group A. These changes increase the intracranial blood volume and intracranial pressure, thereby affecting the balance between cerebral oxygen supply and demand.

**Figure 5:**
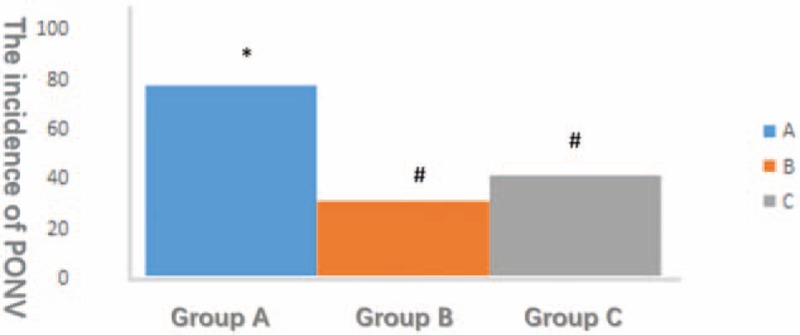
The horizontal axis represents different groups and the vertical axis represents the incidence of PONV. The incidence of PONV in group A is significantly higher than in group B and C (*P* < .05).

The incidence of PONV was much lower in group B and C than in group A and achieved statistical significance (*P* < .05; Fig. 5).

**Figure 6:**
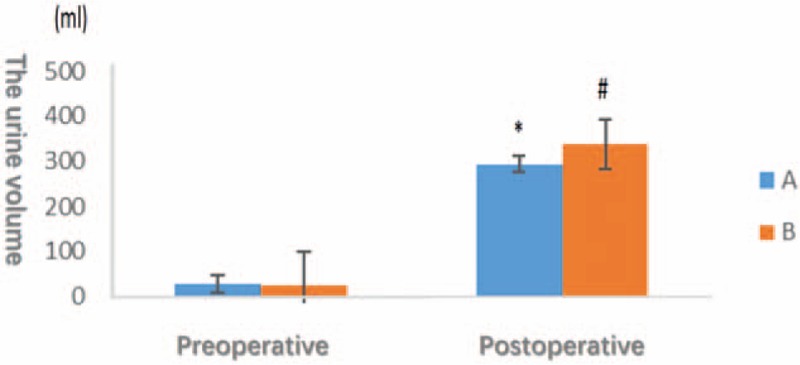
The horizontal axis indicates different times, the vertical axis represents the urine volume of the patients undergoing gynecological endoscopic surgery. There is no significant difference between 2 groups before operative (*P* > .05). The urine volume in group B is significantly higher than in group A after operative.

The preoperative urine volume was not significantly different (*P* > .05), whereas the postoperative urine volume was significantly increased in group B compared with group A (*P* < .05; Fig. 6).

The incidence of PONV was significantly higher in group A than in the other groups. The incidence of PONV was reduced in patients who used mannitol to decrease intracranial pressure by diuresis.

## 4 Discussion

PONV is one of the most common postoperative complications, particularly in gynecological laparoscopic surgery. We assessed the effects of the operative position and artificial pneumoperitoneum on variations perioperative SCTO_2_, RI, and VM to solve this clinical problem and further study the underlying mechanisms and interventions to prevent PONV.

The incidence of PONV was higher in patients who underwent gynecological endoscopic surgery in this study, consistent with the results from a previous study. The reason is related to changes in cerebral tissue oxygen saturation, which may be related to the increase in intracranial pressure caused by pneumoperitoneum and the Trendelenburg position. Second, mannitol reduced the incidence of PONV in these patients by reducing intracranial pressure. Intracranial hypertension was confirmed to be the cause of the higher incidence of PONV in these patients, and mannitol was an effective intervention drug.

According to Apfel et al, the related contributing factors include the following: the female sex, nonsmokers, a history of PONV or motion sickness, and intravenous opioid use.^[3]^ Laparoscopic surgery may increase the systemic vascular resistance and reduce cardiac output,^[4,5]^ subsequently regulating the patient's hemodynamic status to alter end-organ blood perfusion and oxygen delivery. The risk of PONV may be elevated by 60% when the surgery duration is extended by 30 minutes.^[6]^

The pneumoperitoneum mediator CO_2_ may result in increased intracranial pressure, the mechanism may be due to a high pressure-induced impairment in the venous return to the lumbar vertebral venous plexus and cerebral vessel angiectasis induced by hypercapnia.^[7–9]^ The high intracranial pressure leads to cerebrocellular edema, a decrease in oxygen consumption and utilization by the brain tissue, and insufficient cellular oxygenation, thereby triggering the abundant release of inflammatory substances, such as histamine and 5-HT (5-hydroxytryptamine or serotonin), ultimately promoting the occurrence of PONV after gynecological laparoscopic surgery.

SCTO_2_ monitoring reflects a mixture of the SCTO_2_ comprising 70% venous blood and 30% arterial blood. An increased SCTO_2_ indicates an increase in the cerebral venous oxygen content and lower oxygen consumption and metabolic rates. A previous study revealed the relationships between SCTO_2_ and invasive monitoring of carotid sinus oxygen saturation (SjvO_2_).^[10]^ SjvO_2_ was approved by the US Food and Drug Administration (FDA) as a SCTO_2_ monitor^[11]^; hence, evaluations of the brain oxygen status using SCTO_2_ are feasible. The SCTO_2_ value increases as the cerebral blood perfusion or inhaled oxygen concentration increase or as brain metabolism is inhibited, otherwise the SCTO_2_ decreases. We observed an increasing trend that was within the normal range in patients in the 3 groups at T_1_ and T_2_, potentially because pure oxygen was used during anesthesia induction, which increases blood oxygen concentration, and venous general anesthetics decrease cerebral metabolism.^[12]^ The SCTO_2_ was slightly increased and then decreased in groups A, B, and C, but the extent of the increases in groups B and C were relatively small. The following reasons may be responsible for this finding: The use of the Trendelenburg position in groups A and B increased the intracranial pressure due to the gravitational force, subsequently generating cerebral tissue edema, decreased oxygen consumption and utilization, and ultimately increased brain oxygen saturation. Patients in group B received a fast intravenous drip of a small dose of mannitol, which alleviated the intracranial pressure and brain edema via diuresis and further promoted the intake and utilization of cerebral oxygen, reducing the extent of the increase in brain oxygen saturation. SCTO_2_ variations may be related to cerebral vasodilation from peritoneal CO_2_ absorption, but the amplitude of the change was smaller in groups B and C than in group A due to the use of the elevated dorsal position.

The circulation supply in the brain is mainly derived from the vertebral artery of carotid artery system, which provides blood perfusion to the brainstem, cerebellum, lower part of the temporal lobe, and cortex on the inner side of the occipital lobe. The vestibular system, which is located in the brainstem, is extremely sensitive to ischemia, and patients often present symptoms of dizziness, nausea, vomiting, pain in the head or neck, or tinnitus in the presence of an insufficient vertebral blood supply. Our study did not reveal a statistically significant difference in the baseline VM among the 3 groups. After mannitol administration, the VM was obviously faster in group B than in the other 2 groups. We postulated that the fast drip of a small dose of mannitol mitigated the increased intracranial pressure and brain tissue edema, while increasing the blood volume by transferring the interstitial fluid to vessels, which increased the heart stroke volume to accelerate vertebral artery blood flow velocity. The VM variations observed in groups A and C may be related to CO_2_ pneumoperitoneum.

Mannitol alleviates intracranial pressure, increases the brain blood supply, decreases blood viscosity, and improves cerebral oxygenation and microcirculation.^[13,14]^ Intraoperative use of mannitol has been shown to increase the brain oxygen content in patients undergoing long duration retroperitoneal laparoscopic surgery.^[15]^ Preoperative use of a small dose of mannitol (0.5 mg/kg) is safe and may relieve side effects, such as electrolyte disturbances, hypotension and renal failure.^[16]^ Based on our results, the fast intravenous drip of a small dose of mannitol increased the urine volume, reduced SCTO_2_, and increased the brain blood flow, further ameliorating the release of inflammatory mediators, such as histamine and 5-HT, and preventing the occurrence of PONV following gynecological laparoscopic surgery. The possible reason may be the decrease in the intracranial pressure caused by the diuretic effect of mannitol.

In summary, the incidence of PONV was clearly decreased in group B compared with groups A and C. SCTO_2_ and the incidence of PONV were obviously decreased in groups B and C compared with group A. VM was much faster in group B than in group A, indicating that a decrease in SCTO_2_ and an increase in brain blood flow may prevent PONV.

## 5 Conclusions

The high risk of PONV in patients undergoing gynecological laparoscopic surgery may be due to an imbalance in oxygen supply and demand in the brain tissue caused by elevated intracranial pressure. A preoperative intravenous mannitol drip may prevent PONV by reducing the perioperative intracranial pressure.

## 6 Study limitations

Although pneumoperitoneum and the Trendelenburg position can increase intracranial pressure, the direct methods to measure intracranial pressure are currently unavailable. Mannitol was used as a diuretic to decrease the intracranial pressure, indirectly verifying the study hypothesis. Therefore, this study is not quantitative. Only a few studies have explored the correlation between SCTO_2_ and PONV, and thus, more research is needed.
